# Survey of biogenic amines (histamine and spermidine) in commercial seafood by enzyme linked immunosorbent assay (ELISA)

**DOI:** 10.1080/07853890.2021.1896888

**Published:** 2021-09-28

**Authors:** Mário Sousa Diniz, Carolina Madeira, João Paulo Noronha

**Affiliations:** a UCIBIO, Department of Chemistry, Faculdade de Ciências e Tecnologia, Universidade NOVA de Lisboa, Caparica, Portugal; b LAQV-REQUIMTE, Departamento de Química, Faculdade de Ciências e Tecnologia, Universidade Nova de Lisboa, Caparica, Portugal

## Abstract

**Introduction:**

Worldwide there is serious concern about food and consumer safety [1], namely with seafood products. Consequently, there is a major concern regarding food spoilage which make them unsuitable for human consumption. When deteriorating, seafood products suffers a complex series of events that begins when the organism die [1,2]. Therefore, there is a strong need for developing reliable seafood quality analysis. In the present study we surveyed histamine and spermidine in several seafood products (fresh fish and clams), purchased in a Portuguese traditional market.

**Materials and methods:**

Fresh seafood (*Sardina pilchardus*, *Trachurus trachurus*, *Sparus aurata* and *Ruditapes decussata*) were purchased in a market and taken to the laboratory in refrigerated containers. A total of 10 specimens were sampled from each species. Then samples were processed for analysis by homogenising in a phosphate buffer saline solution, centrifuged (10,000×*g* at 4 °C) for 15 min) and then stored at –80 °C until analysis. Seafood samples were assessed for the presence and content of histamine and spermidine using an indirect Enzyme Linked Immunosorbent assay (ELISA) [3]. The statistical analysis was performed using the Mann–Whitney *U*-test to determine differences between biogenic amine levels in seafood samples. Statistics was performed with a significance level of 5%, using the software Statistica 8.0 (Statsoft, Tulsa, OK, USA).

**Results:**

The results show variable results between species (from < LD to 184104 mg histamine/Kg wet weight). The highest levels were detected in T*. trachurus* samples and the lowest in clams. However, it was possible to detect the presence of the selected biogenic amines (histamine and spermidine) in most samples analysed. The lowest levels of spermidine were determined in *R. decussata* (1504.43 mg/kg w.w.), while the highest levels were determined in *T. trachurus* (184104 mg/Kg w.w.). Regarding histamine, the lowest levels were determined in *R. decussata* 20.23 mg/Kg w.w.) and the highest levels were measured in *T. trachurus* (460.25 mg/kg w.w.).

**Discussion and conclusions:**

Although we are capable to detect the presence of the selected biogenic amines, in most of the samples the levels were below the limits established by Food and Drug Administration [4] and the European Union Commission Regulation (EC) No 1441/2007 [5].
